# Nurses’ and nurse leaders’ perspectives on a health-promoting work environment: a meta-ethnographic study

**DOI:** 10.1080/17482631.2025.2460255

**Published:** 2025-01-31

**Authors:** Diako Morvati, Rita Solbakken, Jonas Vaag, Yvonne Hilli

**Affiliations:** a Faculty of Nursing and Health Science, Nord University, Bodø, Norway; b Department of Psychology, Inland School of Business and Social Sciences, University of Inland Norway, Lillehammer, Norway

**Keywords:** Health-promoting, caring sciences, nursing leadership/management, nursing, meta-ethnography, healthy work environment, occupational health

## Abstract

**Purpose:**

The purpose of this meta-ethnography is to integrate and synthesize nurses’ and nurse leaders’ perspectives on a health-promoting work environment to enhance understanding of its essential aspects.

**Methods:**

A meta-ethnographic approach developed by Noblit and Hare was conducted.

**Findings:**

Line of argument synthesis led to the development of an overarching tree metaphor: “cultivating a flourishing environmental tree rooted in values, held stable by leadership, and nurtured by safe working conditions.” This metaphor illustrates that a health-promoting work environment is imbued with three interdependent aspects: 1) core values as the roots of the tree, including respect, recognition, community, and engagement 2) value-conscious leadership as the trunk of the tree, meaning a leader who is conscious of their power position and responsibilities and 3) safe working conditions as fertile soil for the tree, comprising the physical and administrative dimensions of the work environment.

**Conclusions:**

Collaboration between nurses and leaders is crucial for cultivating a health-promoting work environment. However, nurse leaders, due to their influential positions, have the responsibility to facilitate this environment. Consequently, leaders need to receive adequate resources and support from their superiors to foster an environment that enhances nurses’ health and job satisfaction.

## Introduction

The work environment in healthcare has become increasingly stressful due to societal changes in the form of medical, demographic, and technological developments, reimbursement policies, shortages of nurses, and patient complexity (Boniol et al., 2022; Haddad et al., 2023; Phillips et al., 2022). International Council of Nurses (2021) has declared the shortage of nurses a global health emergency, warning of a potential shortfall of up to 13 million nurses by 2030. In addition, many nurses have expressed their intention to leave the profession. Work environmental factors, especially those causing psychosocial strain are associated with nurses’ intentions to leave the workplace (De Oliveira et al., 2017; Heinen et al., 2013). Previous studies highlighted that a lack of a healthy work environment, characterized by factors such as poor working conditions, limited career development opportunities, lack of visible and supportive leadership, ethical and work-related stress, inconsistencies between nursing education and practice, and instances of bullying, are key motivators for nurses leaving the profession (Bahlman van Ooijen et al., 2023; Rosengren & Friberg, 2024). Furthermore, earlier studies emphasized the importance of a HPWE for nurses’ job satisfaction, reducing turnover, stress, burnout, and the retention of healthcare professionals (Akerjordet et al., 2018; Hayward et al., 2016; Mabona et al., 2022; Rosengren & Friberg, 2024) as well as in enhancing practice, quality of care, and patient safety within health and care services (Kieft et al., 2014; Mihdawi et al., 2020; Oshodi et al., 2019).

The importance of promoting employees’ health, well-being, and job satisfaction has led to the development of several models and frameworks, each based on different theoretical perspectives. These include, among others, WHO’s healthy workplace model (WHO, 2010), American Association of Critical-Care Nurses’ six standards for developing a healthy work environment (skilled communication, genuine collaboration, effective decision-making, appropriate staffing, meaningful recognition, and authentic leadership) (2005), effort-reward imbalance model (Siegrist, 1996), job demands-control(−support)-model (Karasek & Theorell, 1990), and job demands resources-model (Demerouti et al., 2001).

This meta-ethnography focuses on the concept of a Health Promoting Work Environment (HPWE) from a caring science theoretical perspective. The literature on HPWE has traditionally employed concepts such as occupational health, healthy workplaces, organizational health, and workplace health promotion in discussions about promoting health within a professional setting (Hanson, 2007; Karlsson, 2010; Orvik & Axelsson, 2012). A setting, according to the World Health Organization (World Health Organization (WHO) (2021), refers to a place or social context where individuals engage in daily activities, in which environmental, personal, and organizational factors interact to affect health and well-being. Workplace health promotion emerged in the 1970s, emphasizing changing individual lifestyles or behaviours (Kuhn & Chu, 2022). Since the 1990s, there has been a shift towards a more interdisciplinary approach to workplace health promotion, driven by an enhanced understanding of the complex factors influencing employee health. This includes transitioning from emphasizing individual-oriented “wellness” activities to a more integrative and holistic setting approach. This evolution reflects a broader recognition of the interplay between various aspects of employee well-being within the work environment (Kuhn & Chu, 2022). For instance, both the WHO (2010) and European Network for Workplace Health Promotion European Network for Workplace Health Promotion (ENWHP) (1997) describe a healthy workplace as an environment where employers, employees, and society work together in a continuous improvement process to protect and promote the health, safety, and well-being of all workers. This approach emphasizes the importance of involving both employees and leaders/manager in a collaborative process to develop a HPWE (Kuhn & Chu, 2022).

In leadership research, the terms “nurse leader” and “nurse manager” are often used interchangeably (Cummings et al., 2021; Weiss et al., 2023). In this study, “nurse leader” is used, which refers to individuals with a nursing background in mid-level leadership positions, responsible for both personnel and patient care. Furthermore, leadership and management are viewed as distinct but complementary activities: leadership focuses on human and relational aspects, while management involves facilitating, organizing, and planning the conditions that enable successful leadership (Alvesson & Spicer, 2012; Vevatne et al., 2020. Previous studies have highlighted the influence of nurse leaders on the nursing work environment, particularly the significant relationship between nursing leaders’ leadership style and nurses’ health, job satisfaction, and well-being (Cummings et al., 2018; Kohnen et al., 2024; Niinihuhta & Häggman-Laitila, 2022). Collaboration between nurses and nurse leaders is essential for fostering a HPWE (Jiménez et al., 2017; Mabona et al., 2022; Wei et al., 2018). Therefore, it is vital to integrate both perspectives.

Evidence-based decision-making in healthcare services requires more synthesis of qualitative research to gain a deeper understanding of people’s experiences, which can holistically guide practical interventions (France et al., 2019; Uny et al., 2017). While there is a body of qualitative research on HPWE, no qualitative synthesis exists that integrates the experiences and perspectives of both nurses and nurse leaders regarding HPWE. To address this knowledge gap, we seek to synthesize both perspectives on HPWE—an environment that promotes health and well-being among nursing staff. To achieve this goal, we have chosen a meta-ethnographic approach (Noblit & Hare, 1988) because it enables the synthesis of qualitative findings to construct new and overarching insights that go beyond the results of individual studies (France et al., 2019; Noblit & Hare, 1988). This method is suited for exploring the dynamics of HPWE, as it integrates the shared experiences of nurses and nurse leaders to enhance understanding of the essential aspects of a HPWE.

## Theoretical perspective

The understanding and description of health directly impact how the concept of a HPWE is perceived or interpreted (Torp, 2013). The theoretical perspective is based on a caring science perspective, in which caring is seen as the essence of nursing. From this perspective, health is understood as more than the absence of disease; it encompasses physical, spiritual, mental, and social well-being. It focuses on the individual’s health resources and potential, and the impact of environmental and contextual factors on an individual’s health (Eriksson, 2018). Health is a dynamic process characterized by a movement towards realizing the individual’s potential, and towards a greater whole (Eriksson, 2018). In the most profound sense, health is an ontological concept linked to the process of becoming. Health means being whole, integrated, and holy, and to experience holiness, one must be in touch with one’s innermost core or ethos (Eriksson, 2018). Ethos is the person’s basic values that promote the good life, where a person feels metaphorically “at home”. The home as the ethos comprises three dimensions. The innermost dimension is imbued with a person’s basic values or ethos. The middle dimension encompasses a person’s manner of being an ethical code of conduct, all shaped by the ethos and reflected in the third, physical, and outermost dimensions, where persons meet, act, and interact (Hilli, 2007; Hilli & Eriksson, 2019). Individuals who are in touch with their ethos are internally driven, living in freedom and harmony with themselves and their surroundings. They are committed to spreading joy, respect, and inspiration to those around them. When many people, in this case, in a work environment share the same ethos, they collectively create an environment where everyone can feel metaphorically at home, a place where it is good to be (Hilli, 2007).

## Purpose

The purpose of this meta-ethnography is to integrate and synthesize nurses’ and nurse leaders’ perspectives on a health-promoting work environment to enhance understanding of its essential aspects.

## Methods

### Design

In this study, a meta-ethnographic approach described by Noblit and Hare (1988) was chosen. Meta-ethnography, initially developed as a basis for meta-synthesis, is a widely used approach in nursing research (Bondas & Hall, 2007). Meta-ethnography aims to interpret and synthesize the findings from qualitative primary studies to develop new theories, syntheses, and understandings of a concept or phenomenon. Synthesis involves interpretation rather than description. Therefore, meta-ethnography is more than summarizing and aggregating (France et al., 2019; Noblit & Hare, 1988). Noblit and Hare’s method consists of seven phases: (1) getting started, (2) deciding what is relevant to the initial interest, (3) reading the studies, (4) determining how the studies are related, (5) translating the studies into one another (6) synthesizing translations and (7) expressing the synthesis. Additionally, the eMERGe reporting guidance developed by France et al. (2019) was followed to enhance transparency in the reporting of this meta-ethnography (Supplemental File1).

### Data collection and synthesis

#### Phase 1— getting started

Our interest in conducting this meta-ethnography arises from an identified knowledge gap: the need for a qualitative synthesis that integrates and synthesizes the perspectives of both nurses and nurse leaders regarding HPWE.

#### Phase 2—deciding what is relevant

In phase 2, search strategies were developed with guidance from a senior librarian. The first author conducted a pilot search on Google Scholar to identify relevant keywords in similar studies. Additionally, the authors held a collaborative meeting to discuss the selection of relevant databases and the criteria for inclusion and exclusion, as detailed in Table I.

**Table I.** Inclusion and exclusion criteria.

Inclusion criteria	Exclusion criteria
Peer-reviewed primary qualitative studies of all qualitative methodologies, published in scientific journals	Quantitative studies
Scandinavian or English language	Language other than Scandinavian or English
Focused on the perspectives of nurses or nurse leaders with or without any continuing education in any kind of clinical setting.	Studies that do not focus on the perspectives of nurses or nurse leaders.
Studies with mixed participants or mixed methods where qualitative findings from the nurse’s or nurse leader’s perspective could be distinguished and identified.	Theoretical and review studies

Search strategies were adapted to each database using a combination of these keywords: (“health promotion” OR “health-promoting” OR organizational health” OR “promotion of health”) AND (“work environment” OR “working conditions” OR “psychosocial work environment” OR “workplace” OR “workplace health”) AND (nurse* OR “nurse manager” OR “nurse leader” OR nurse administrator) AND (qualitative research OR interview*OR qualitative study OR qualitative*). A systematic search for relevant studies, without year or geographical limitations, was conducted by the first author between January and June 2023 in the following databases: Medline (including PubMed), CINAHL, SCOPUS, PsycINFO (Ovid), Science Direct, and Web of Science (Supplemental File 2). The systematic search resulted in 3,658 records, with 3,585 identified through databases and 73 identified via manual searches of the reference lists of included articles. Duplicates were removed, and the number of studies was reduced to 2720. After reviewing the title and abstract, 73 studies were selected for further assessment.

**Figure 1.**
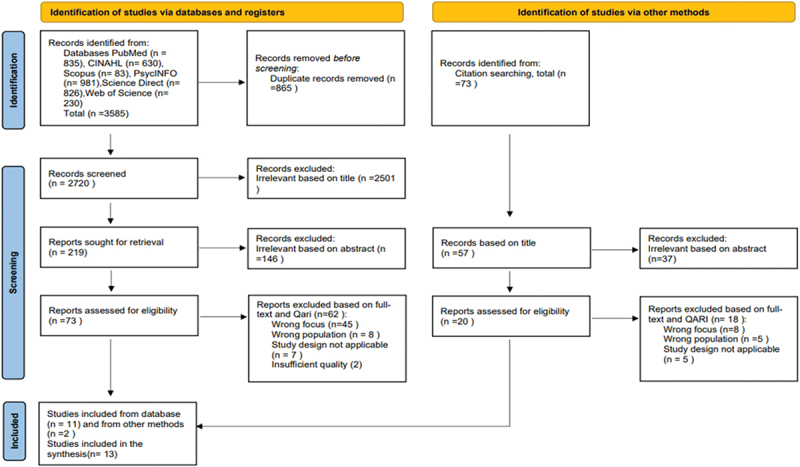
Prisma flow diagram (Page et al., 2021).

Based on reading the full text of the studies and assessing the relevance of their findings to HPWE, as well as adherence to inclusion criteria, 15 studies were initially deemed suitable for inclusion in this meta-ethnography. The Joanna Briggs Institute (JBI) Qualitative Appraisal and Review Instrument (QARI) (Lockwood et al., 2015) was used to critically appraise the 15 studies that met the inclusion criteria. However, after a quality assessment, two studies were excluded, resulting in a final inclusion of 13 studies. In the quality assessment of the studies, we agreed to have more focus on conceptual richness that can shed light on a HPWE, while ensuring that the reported methods are of sufficient quality (Supplemental File 3). An overview of the search process is illustrated in Figure 1.

The included studies were published between 2009 and 2023 and involved 476 participants, of which 311 were nurses and 165 were nurse leaders/managers. The gender distribution among the participants was 68 males, 389 females, and 1 transgender individual. Two included studies did not report on the participants’ gender (Furunes et al., 2018; Hartung & Miller, 2013). The 13 included studies represent six countries: USA (5), Norway (2), Sweden (2), Iran (2) Nepal (1), and Turkey (1). Of the included studies, seven were from the nurse’s perspective, three were from the leader’s perspective, and three were from both perspectives, where it was possible to distinguish between nurses’ and leaders’ perspectives. Gadolin et al. (2022) study used a mixed-method approach. It was included since it met the inclusion criteria, and we extracted and focused on the qualitative findings. The characteristics of the included studies are presented in Table II.

**Table II.** Characteristics of the included studies.

Author, Year, Country	Context	Aim	Design and methods: - Data collection -Participants (male/ female) -Data analysis method	Key concepts
(Khalafi et al., 2023) Iran	Seven different hospital anesthesia departments in Ahvaz	To explain the factors affecting the workplace health of Iranian anesthesia teams.	A phenomenology design -Individual in-depth semi-structured interviews −14 nurse anesthetists (5/9) −3 chief nurse anesthetist (1/2) -Qualitative content analysis	Teams work Managers need to meet nurses’ physical and mental needs Communication Role clarify Adequate supervision Coworker trust Interpersonal justice Workplace accommodations Availability of equipment
(Thapa et al., 2022) Nepal	Hospitals in Kathmandu Valley, Nepal-	To explore and thereby gain a deeper understanding of how nurses in Nepal’s hospitals experience their everyday work, with a focus on promoting and sustaining their work-related health.	Qualitative design - Individual in-depth Semi-structured interviews −19 female nurses -Thematic analysis.	Sense of meaningfulness and belongingness Open environment and sharing Cooperation across the entire team Emotional support Support and rewards from the management Fair evaluation and job promotion opportunities Being acknowledged, appreciated, and rewarded. A manageable workload Feeling safe from work-related hazard Skills development training and educational training Activities outside of work needed for recovery
(Gadolin et al., 2022) Sweden	Medical specialties, in six different hospitals	How healthcare unit managers act and organize their work to promote nurses’ perceived organizational support and hence to ensure nurses’ health and well-being.	Mixed methods design comprising qualitative interviews and quantitative surveys - Individual in-depth semi-structured interviews 20 healthcare unit nurse managers (2/18) -Qualitative content analysis	The need to be available for nurses Opportunities to acknowledge and support individual team members Facilitate the development of trust within the work team Always having an “open door” for the staff Providing supervision and mentorship and education and training Created a sense of security Good collegial cooperation Information Managerial trust, communication, participation, and autonomy Safeguarding a sound working environment Collegial dialogue Sharing of experience
(Thapa et al., 2021) Sweden	Hospitals and community healthcare facilities	To explore and gain a deeper understanding of how nurses and midwives experience their everyday work, to promote and sustaining their work-related health	Qualitative descriptive design - Individual in-depth semi-structured interviews − 13 nurses (2/11) -Qualitative content analysis	Supportive environment Social and collegial teamwork Suppurative management Present and responsive manager To be valued and acknowledged Valuable work-related information Sufficient personnel Adequate instructions Competence opportunities for job satisfaction Supervision and reflection Importance of collegial support and teamwork Social activities both within and outside the workplace
(Johansen et al., 2021) USA	5 hospitals.	To explore how acute care clinical nurses and nurse managers perceived their healthy work environment.	Qualitative design - Focus Groups interviews −89 participants: clinical nurses = 46 (4/42) and nurse managers = 43 (9/33 and 1 transgender) -Thematic analysis	Mutual respect Communication Collaboration- interdisciplinary and interdepartmental collaboration Teamwork Effective decision making Appropriate staffing People want to be acknowledged Leadership- to be present and approachable Mentorship
(Oneal et al., 2019) USA	Acute care hospitals and other healthcare contexts (clinic, rehabilitation facility/nursing home, home care)	This study examined factors related to the overall work, safety, and the health of nurses that should be addressed in work environments to promote well-being and prevent burnout and attrition.	Qualitative descriptive design - Focus group and individual interviews − 34 newly licensed nurses participated (7/27). - Thematic analysis	Attitudes and respect for others Good role modelling and support from their mentors or managers Safe environment Communication between staff Organizational support Resources- physical room, equipment, human resources Learning to be- development of a professional nursing practice Self-autonomy and self-advocacy Recognition and understanding of nurse’s challenging
(Samur & Seren Intepeler, 2019) Turkey	Internal disease, gastrology, endocrinol‐ ogy, anesthesia intensive care, and cardiology clinics of a large university hospital in Izmir	To gain a deep understanding of the nurses’ views of their work environment	Qualitative descriptive design - In-depth individual interviews − 17 nurses (1/16) - Thematic analysis	Room structures and plans illumination Hygiene condition- a clean and hygienic work environment Information The attitude of managers Team collaboration Communication
(Furunes et al., 2018) Norway	Home care nursing	To increase knowledge about nurses’ understanding of a health‐promoting work environment and health‐promoting leadership.	Qualitative descriptive design - Individual in-depth semi-structured interviews −12 experienced registered nurses - Qualitative content analysis	Autonomy Role clarity Job demands Participation in decision-making Skills and competence development Social support. Health‐promoting leaders should be attentive and take action
(Huddleston & Gray, 2016) USA	Acute Care Settings	To explore the nurse leaders’ and direct care nurses’ perceptions of of a HWE, and to define the characteristics of a HWE in acute care settings.	-Qualitative exploratory descriptive design - Focus group interviews −129 participants: 72 nurse leaders (12/60) and 57 direct care nurses (8/49). - Qualitative thematic analysis	Respect and trust Appropriate staffing Enough time Safe environment Authentic leadership Decision making Recognition Communication Feedback Collaboration Teamwork Physical and psychological safety
(Atefi et al., 2014) Iran	Surgical, medical, and critical care wards of a large hospital	To explore factors related to critical care and medical-surgical nurses’ job satisfaction as well as dissatisfaction in Iran	Qualitative descriptive design - Focus group interviews − 85 nurses (11/74 females) -Thematic analysis	Involvement in patient care Teamwork- working with supportive and helpful colleagues Benefit and reward Working condition Access to medical resources Role clarify Importance of leadership skills- supportive and cooperative- acknowledge their staff’s work Enough time to give patient care. Professional development Clinical autonomy
(Hartung & Miller, 2013) USA	Care center-patient care setting: inpatient and outpatient units	This study focuses on nurse managers’ perceptions of communication and healthy workplace.	Qualitative design - Individual in-depth interviews − 6 nurse managers -Qualitative data analysis	Communication Promoting positivity Openness, open environment Information To be available for the staff Being an example Staff empowerment Shared governance Encouraged staff to communicate with each other Giving feedback
(Averlid & Axelsson, 2012) Norway	6 different hospital anesthesia departments in the southeast region of Norway.	To explore different factors that may contribute positively or negatively to the work environment of nurse anesthetists in Norway	- Qualitative design- grounded theory - Individual interviews − 14 nurse anesthetists (6/8) - Systematic text condensation	Physiological and psychological Needs Enough time Collaboration Trust Teamwork Feedback Open environment Personal and professional learning and development Communication Appreciation from managers and colleagues Mentorship Suppurative Culture
(Shirey, 2009) USA	Acute care hospitals	To showcase the relationship among authentic leadership, organizational culture, and healthy work environments	Qualitative descriptive design - Individual interviews − 21 nurse managers (all females) - Qualitative data analysis	Communication Having somebody that I share values with Staff meetings Collaboration Recognition Teamwork Nurses should be involved contributors to key decision Appropriate staffing Authentic leadership Promoting core values

#### Phase 3—reading the studies

In this phase, we initiated the synthesis process by thoroughly reading the included studies to become as familiar as possible with the key concepts (Noblit & Hare, 1988). Since the included studies involved perspectives from nurses and leaders, the first author created separate tables for each perspective/group. Subsequently, the “raw data” was extracted from each perspective, encompassing first-order constructs (participant quotations) and second-order constructs (primary study’s author interpretations). All authors first individually read the articles and raw data. Subsequently, we collectively discussed how the different studies could be related to each other.

#### Phase 4—determining how the studies are related

Noblit and Hare (1988) outline three possible ways to synthesize: (1) reciprocal: when studies can be compared because they are sufficiently similar in their focus, (2) refutational: when studies are in opposition to each other, and (3) line of argument which aims to make a whole into something more than the parts alone imply (Noblit & Hare, 1988). A line of argument synthesis can be a useful way to bring together and synthesize perspectives of two or more different groups, in this case, nurses and nurse leaders’ perspectives, and interpret the relationship between their understanding of the phenomenon of interest (Sattar et al., 2021). One or all three of these strategies may be used in meta-ethnography (France et al., 2019). We decided first to conduct a reciprocal synthesis within each perspective separately, as the findings from each were comparable. Thereafter, we developed a line of argument from both perspectives, as they were not contradictory but rather analogous in their views on a HPWE.

#### Phase 5—translating the studies into one another

In this phase, the first author translated and integrated common concepts from the primary studies within each perspective into each other. Specifically, we created two separate tables: one for the studies from the nurses’ perspectives and one for the leaders’ perspectives. Subsequently, the first author translated the integrated common concepts, leading to the development of themes that provide a deeper and holistic insight into the concept of HPWE. The translation is characterized by idiomatic rather than literal rendering. It involves taking findings from one study and incorporating them into another, even if they are not expressed verbatim but convey the same underlying meaning (Noblit & Hare, 1988).

#### Phase 6—synthesizing translations

As we mentioned in phase 4, the findings from the perspectives of nurses and nurse leaders were not contradictory; rather, they were analogous and comparable. Therefore, in this phase, we conducted a line of argument synthesis integrating findings from the nurses’ and nurse leaders’ perspectives to develop a holistic and profound understanding of the concept of HPWE. Through an iterative and dialectic movement between the whole and the parts, we continuously alternated between the findings of the primary studies from both perspectives, leading to the development of a deeper understanding and interpretation. This movement led to the creation of a “tree” metaphor, illustrating third-order constructs of the concept of a HPWE.

## Ethical considerations

Although human participants were not involved in this study, ethical considerations were addressed in the included studies. All included studies mentioned that they had received ethical approval from a research ethics committee.

## Findings

To express our findings, we first present the findings from each perspective—nurses and nurse leaders. Thereafter, we present findings from the line of argument synthesis, where we synthesized both perspectives to enhance understanding of the essential aspects of a HPWE.

### Nurse’s perspective on a health-promoting work environment

From the nurses’ perspectives, a health-promoting work environment is imbued with six aspects: 1) an environment of mutual respect and recognition, 2) a sense of togetherness in the workplace 3) learning and having autonomy in daily work 4) having a responsible and supportive leader, 5) importance of balance between demands and resources, and 6) feeling safe in the workplace.

#### An environment of mutual respect and recognition

The nurses expressed that being acknowledged, treated fairly, and having relationships based on mutual trust, where each acts and interacts respectfully with others, are crucial aspects of a HPWE (Atefi et al., 2014; Huddleston & Gray, 2016; Johansen et al., 2021; Thapa et al., 2022). Being treated fairly could, among others, mean receiving equal opportunities and support irrespective of one’s background (Khalafi et al., 2023; Thapa et al., 2021). Recognition of each other’s competence and contributions was described as one way of showing respect and acknowledging each other; nurses must believe their skills and knowledge are valued and their efforts are not overlooked (Atefi et al., 2014; Furunes et al., 2018; Huddleston & Gray, 2016; Johansen et al., 2021; Oneal et al., 2019; Thapa et al., 2021). Further, the nurses emphasized the importance of respecting others’ meanings and opinions and providing opportunities for individuals to feel comfortable expressing their opinions and values about their work situation (Averlid & Axelsson, 2012; Huddleston & Gray, 2016; Johansen et al., 2021; Samur & Seren Intepeler, 2019; Thapa et al., 2021).

#### A sense of togetherness in the workplace

A sense of togetherness emerges as a critical element in a HPWE, wherein employees actively seek opportunities to cultivate a community where they collaborate, share knowledge, and support one another in attaining common goals (Atefi et al., 2014; Averlid & Axelsson, 2012; Huddleston & Gray, 2016; Thapa et al., 2022). A sense of togetherness and support from colleagues, according to nurses, results in reduced stress, promotes happiness and job satisfaction, and ultimately strengthens their health (Atefi et al., 2014; Furunes et al., 2018; Oneal et al., 2019; Thapa et al., 2021, 2022). The nurses further emphasize the importance of engaging in social activities outside of work as significant contributors to enhancing team bonding. These activities extend beyond the workplace and provide opportunities for team members to connect on a personal level, fostering stronger relationships and a sense of togetherness and community (Atefi et al., 2014; Thapa et al., 2021, 2022).

#### Learning and having autonomy in daily work

The nurses underscored the importance of having autonomy and opportunities for learning in the workplace, clear job descriptions, and defined responsibilities within their roles and competencies. These factors are vital to nurses’ feeling of development and mastery in the workplace (Atefi et al., 2014; Furunes et al., 2018; Huddleston & Gray, 2016; Oneal et al., 2019; Thapa et al., 2022). Skills and educational development are essential for nurses’ job satisfaction and well-being. Job satisfaction, in turn, enhances nurses’ feeling of safety and their ability to handle different situations (Atefi et al., 2014; Averlid & Axelsson, 2012; Furunes et al., 2018; Huddleston & Gray, 2016; Thapa et al., 2022). Another critical factor that fosters nurses’ experience of autonomy is their participation in the decision-making process, especially in matters that directly affect their work. Many nurses find it motivating to be included in change processes, as it can make their work feel more manageable (Furunes et al., 2018; Huddleston & Gray, 2016; Johansen et al., 2021). An important requirement for nurses to participate in decision-making is accessibility to information. Therefore, nurses emphasize the importance of relevant and timely information in involving employees in workplace matters and preventing conflicts and misunderstandings (Samur & Seren Intepeler, 2019; Thapa et al., 2021).

#### Having a responsible and supportive leader

Leaders, due to their power positions, influence the work environment through standardized guidelines and bureaucratic control over daily practices. Therefore, the nurses view leaders as crucial contributors to the development of a HPWE. They emphasize the importance of leaders being supportive, authentic, visible, responsive, and accessible (Atefi et al., 2014; Averlid & Axelsson, 2012; Furunes et al., 2018; Huddleston & Gray, 2016; Thapa et al., 2021, 2022). According to the nurses, a responsible and supportive leader acts as a good role model, being aware of their attitude and behaviour. They should be fair and attentive to employees’ conduct and interactions, aiming to promote a healthy and respectful environment while also striving to prevent bullying and discrimination (Furunes et al., 2018; Huddleston & Gray, 2016; Johansen et al., 2021; Khalafi et al., 2023; Thapa et al., 2022).

#### Importance of balance between demands and resources

The nurses highlighted the importance of balancing job demands and resources as a paramount aspect of an HPWE. Job resources include, among others, adequate staffing, mentoring, collegial support, feedback, and sufficient time for patient care and administrative tasks (Atefi et al., 2014; Averlid & Axelsson, 2012; Furunes et al., 2018; Huddleston & Gray, 2016; Khalafi et al., 2023). Insufficient staffing, multitasking, a heavy workload, and time constraints can make nurses feel incapable of providing sufficient patient care. Consequently, this can lead to a decrease in nurses’ self-efficacy and collegial cooperation, which can have adverse effects on job satisfaction and their health (Atefi et al., 2014; Furunes et al., 2018; Huddleston & Gray, 2016; Oneal et al., 2019; Thapa et al., 2021, 2022). For some nurses, time pressure leads to feelings of irritation, frustration, and tiredness, as they may perceive that they have not been able to get a good job or meet their values and standards of care (Atefi et al., 2014; Huddleston & Gray, 2016; Thapa et al., 2021, 2022).

#### Feeling safe in the workplace

Another critical aspect of a HPWE is feeling safe from work-related hazards. On one hand, it involves having access to the necessary medical equipment to, for example, protect both themselves and patients from infections. The shortage or malfunction of medical equipment was described as an issue that can affect nurses’ health and job satisfaction (Atefi et al., 2014; Averlid & Axelsson, 2012; Huddleston & Gray, 2016; Khalafi et al., 2023; Thapa et al., 2022). On the other hand, feeling safe involves being provided with appropriate, suitable, and well-designed spaces and rooms for nurses to work comfortably. This includes ensuring a clean and hygienic work environment with adequate width, proper illumination, and sufficient ventilation- and heating systems, all of which are important factors that affect the nurse’s health (Atefi et al., 2014; Averlid & Axelsson, 2012; Oneal et al., 2019; Samur & Seren Intepeler, 2019; Thapa et al., 2022) Furthermore, the findings show that safe working conditions encompass ensuring an environment that protects nurses from both physical and psychological harm, especially when they are confronted with angry patients and relatives (Huddleston & Gray, 2016; Khalafi et al., 2023; Oneal et al., 2019).

### Nurse leaders’ perspective on a health-promoting work environment

From the perspective of nurse leaders, a health-promoting work environment is imbued with four aspects: 1) importance of collaboration, 2) facilitating empowering employees, 3) being an accessible and responsive leader, and 4) maintaining adequate staffing.

#### Importance of collaboration

The nurse leaders emphasized the importance of collaboration and accepting each other as equal members establishing a team to foster collegial support, as these factors contribute to a HPWE and result in the best outcomes for patients, nurses, and the organization as a whole (Gadolin et al., 2022; Hartung & Miller, 2013; Khalafi et al., 2023). Collaboration and collegial support lead to the maintenance and development of trust in the workplace. This fosters cohesion and an experience that everyone is working together towards common goals, ultimately reducing feelings of isolation and pressure, which in turn alleviates stress (Huddleston & Gray, 2016; Johansen et al., 2021; Khalafi et al., 2023).

#### Facilitating empowering employees

Another aspect of a HPWE, is empowering employees by creating environments that enable nurses to take ownership of their roles and professional growth (Gadolin et al., 2022; Hartung & Miller, 2013; Shirey, 2009). Nurse leaders understand that empowerment involves more than just delegating tasks; it entails providing the necessary support and resources for nurses to thrive. Central to facilitating empowerment is the provision of ongoing education and training opportunities, as well as supervision and mentorship (Gadolin et al., 2022; Hartung & Miller, 2013; Huddleston & Gray, 2016; Johansen et al., 2021; Shirey, 2009). These contributions not only enhance nurses’ skills and knowledge but also foster a culture of continuous learning and enhance nurses’ job satisfaction and the quality of patient care (Gadolin et al., 2022; Hartung & Miller, 2013; Shirey, 2009). Furthermore, nurse leaders recognize that it is important to give nurses a voice in matters and involve them in decision-making processes that affect their work (Gadolin et al., 2022; Hartung & Miller, 2013; Huddleston & Gray, 2016).

#### Being an accessible and responsive leader

Leaders underscore the need to be accessible to nurses in their daily work on the unit, as it provides opportunities to be present with employees and support them through direct communication (Gadolin et al., 2022; Hartung & Miller, 2013; Huddleston & Gray, 2016; Shirey, 2009). They emphasized the importance of receiving and providing feedback, as well as maintaining an open-door policy that encourages discussion and transparency (Hartung & Miller, 2013; Huddleston & Gray, 2016; Khalafi et al., 2023). Leaders understand that their influence is best exerted through engagement rather than control or punishment, fostering a work environment of trust and collaboration within the team. Engagement means being recognized, actively listening, giving clear direction, providing, and receiving feedback, and acknowledging employees’ efforts (Gadolin et al., 2022; Hartung & Miller, 2013; Shirey, 2009). Additionally, they emphasize the importance of following up on raised issues and redirecting negativity to maintain focus on the collective goals of the team. This proactive approach ensures that challenges are promptly addressed, and the team remains aligned and motivated to achieve their goals (Hartung & Miller, 2013; Shirey, 2009).

#### Maintaining adequate staffing

Nurse leaders highlighted the importance of maintaining appropriate staffing levels and aligning patients’ needs with nurses’ competencies. However, they pointed out that maintaining adequate staffing levels can sometimes be challenging as they are under pressure for efficiency and financial savings (Hartung & Miller, 2013; Huddleston & Gray, 2016; Johansen et al., 2021; Khalafi et al., 2023; Shirey, 2009). They recognize that heavy workloads and time pressure, because of a lack of adequate staffing, are the main barriers to establishing a healthy work environment. Therefore, they emphasize the need to ensure adequate staffing levels, allocate resources effectively, and implement strategies to mitigate workload pressures for the working environment to be health-promoting (Gadolin et al., 2022; Hartung & Miller, 2013; Huddleston & Gray, 2016; Khalafi et al., 2023; Shirey, 2009).

### Line of argument synthesis of nurses’ and nurse leaders’ perspectives

**Figure 2.**
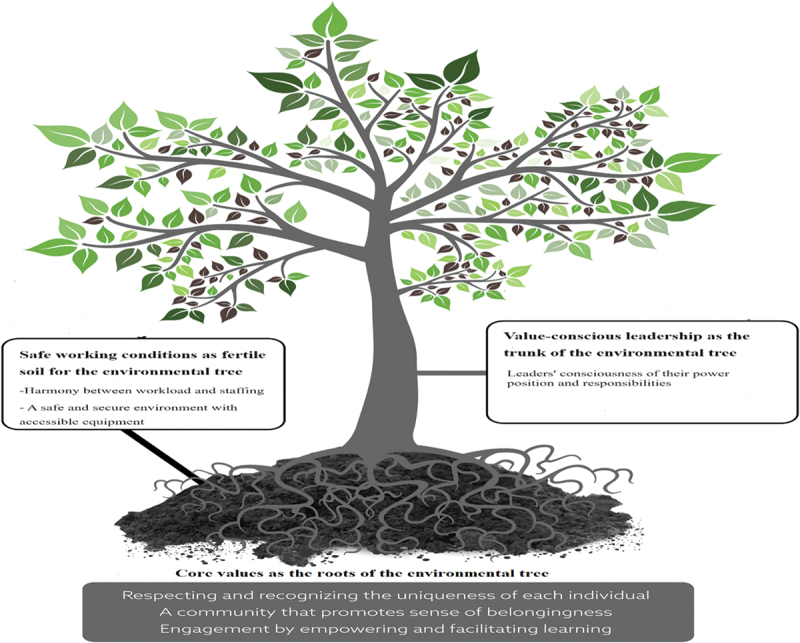
The tree metaphor which illustrates the essential aspects of a health-promoting work environment.

The line of argument synthesis of both perspectives, led to the development of an overarching metaphor: *cultivating a flourishing environmental tree rooted in values, held stable by leadership, and nurtured by safe working conditions*. This metaphor emerged from three themes: 1) core values as the roots of the environmental tree; 2) value-conscious leadership as the trunk of the environmental tree; and 3) safe working conditions as the fertile soil for the environmental tree (Figure 2).

#### Core values as the roots of the environmental tree

The findings show that just as the root is essential for a tree’s growth by providing the foundation for a tree’s survival, the core values establish fundamental character, culture, and spirit within the working environment. The core values comprise respecting and recognizing the uniqueness of each individual, a community that promotes a sense of belongingness and engagement by empowering and facilitating learning. According to the findings, the core values of respect and recognition are considered the foundation of a HPWE. When nurses experience being seen and respected for their importance as a unique person, and when they don’t feel easily replaceable, it can have a positive impact on their overall health and well-being (Atefi et al., 2014; Khalafi et al., 2023; Oneal et al., 2019). A health-promoting work environment is, furthermore, imbued with an atmosphere in which individuals have a shared willingness to cooperate and collaborate as a team (Atefi et al., 2014; Averlid & Axelsson, 2012; Gadolin et al., 2022; Hartung & Miller, 2013; Huddleston & Gray, 2016; Kieft et al., 2014; Thapa et al., 2022). The core value of engagement by empowering and facilitating learning emphasizes the importance of autonomy and professional development in fostering a HPWE. The provision of ongoing learning opportunities contributes to a sense of empowerment among nurses, allowing them to feel valued and supported in their roles. This empowerment fosters a positive work environment where nurses are encouraged to continuously improve their skills and knowledge, leading to increased job satisfaction (Atefi et al., 2014; Furunes et al., 2018; Huddleston & Gray, 2016; Thapa et al., 2021). Another critical factor that fosters nurses’ engagement is shared governance. This emphasizes the significance of involving nurses in decision-making processes related to various aspects of their work, including policy-making, clinical care direction, evaluation, and organizational leadership (Furunes et al., 2018; Gadolin et al., 2022; Huddleston & Gray, 2016; Johansen et al., 2021).

#### Value-conscious leadership as the trunk of the environmental tree

The findings highlight that value-conscious leadership acts metaphorically as the trunk of the environmental tree, connecting the roots (core values) to the branches (employees and various tasks). This leadership provides support and direction to the entire organization, guided by the core values, ensuring a HPWE, much like a sturdy trunk that supports the growth of branches in a tree. This theme comprises leaders’ consciousness of their power position and responsibilities. Nurse leaders, due to their powerful position, have a special responsibility for decision-making, acting as role models, and promoting core values in the workplace. Therefore, nurse leaders’ consciousness of their purpose, motives, value, and behaviour towards their staff is crucial for developing a HPWE (Furunes et al., 2018; Hartung & Miller, 2013; Huddleston & Gray, 2016; Oneal et al., 2019; Samur & Seren Intepeler, 2019; Thapa et al., 2022).

#### Safe working conditions as fertile soil for the environmental tree

Based on the findings, safe working conditions symbolize the fertile soil that provides sustenance and the essential external structures and conditions for employees to flourish. This includes the physical environment, administrative processes, and organizational practices that contribute to the safety and security of the staff. This theme includes harmony between workload and staffing, and a safe and secure environment with accessible equipment. A safe and a HPWE is characterized by maintaining a balance between the number of patients and the number of staff members, as well as between complex patient needs and nurse competencies (Atefi et al., 2014; Averlid & Axelsson, 2012; Furunes et al., 2018; Gadolin et al., 2022; Huddleston & Gray, 2016; Khalafi et al., 2023; Shirey, 2009). Furthermore, the findings emphasize the importance of access to necessary medical equipment and underscore the need for environments that safeguard nurses from both physical and psychological harm, particularly when dealing with challenging situations involving patients and relatives (Atefi et al., 2014; Averlid & Axelsson, 2012; Huddleston & Gray, 2016; Oneal et al., 2019; Samur & Seren Intepeler, 2019; Thapa et al., 2022).

## Discussion

This meta-ethnography aimed to integrate and synthesize nurses’ and nurse leaders’ perspectives on a health-promoting work environment to enhance understanding of its essential aspects. The metaphor of *cultivating a flourishing environmental tree rooted in values, held stable by leadership, and nurtured by safe working conditions*, provides a holistic and dynamic perspective with a strong focus on the moral and value dimension of a HPWE. In such an environment, as metaphorically depicted with the green leaves, nurses will flourish and thrive. Flourishing in the workplace means experiencing well-being and contentment, performing at one’s best, and finding meaning in one’s work (Arakelian & Rudolfsson, 2023). According to Steele et al. (2022) using metaphors in the presentation of findings makes them clearer, helping social phenomena become easier to understand and work with. Metaphors stimulate imagination, evoke emotions, reveal new meanings, and can even inspire change. Using the tree metaphor, we aimed to illustrate the close interconnection between core values, leadership, and safe working conditions, all contributing to the shared vision of cultivating a flourishing and health-promoting work environment. If any of these components are in poor condition, it can negatively affect the entire environment.

According to our findings, a HPWE is imbued with a set of core values or ethos, including respect, recognition, community, and engagement. These core values are inherently linked to factors that promote the health of employees, aligning with WHO’s healthy workplace model (WHO, 2010). Guillemin and Nicholas (2022) highlight that work rooted in core values provides an experience of meaning and aligns with the individual and the values that drive people’s lives. Individuals who are in touch with their ethos, metaphorically feeling “at home,” are internally driven, maintaining integrity, authenticity, and a sense of rootedness in their ethos, which is crucial in promoting their health and well-being (Hilli & Eriksson, 2019). Ethos is the core of caring, encompassing respect for human dignity and integrity, the courage to take responsibility for others, and a commitment to alleviating human suffering (Eriksson, 2018; Fagerström et al., 2021).

Our findings show that daily communication and interactions between employees and leaders strengthen the roots of a HPWE. This means that HPWE is considered a shared responsibility carried by all members of the organization and that everyone plays a part in its development, which is consistent with previous studies (Jiménez et al., 2017; Mabona et al., 2022; Solbakken et al., 2021; Wei et al., 2018). According to Hilli and Eriksson (2019), all human beings carry an ethos which sets the tone in the outer room, in terms of the spirit of the house. When many people, in this case, in a work environment share the same ethos, they collectively create an environment where everyone can feel metaphorically at home (Hilli, 2007; Hilli & Eriksson, 2019).

As in earlier studies, our findings demonstrate that respectful behaviour and interactions in the nursing workplace contribute to the promotion of a caring culture and community (Emeghebo, 2012; Morvati & Hilli, 2023), while disrespect is one of the factors contributing to dissatisfaction among nurses in their work environment (Nouri et al., 2019). A negative environment characterized by a lack of respect, avoidance, and exclusion can lead to feelings of homelessness and rootlessness (Gabrielsen et al., 2014).

Humans strive to be unique, while also longing for relationships with others where they can experience meaning in the sense of becoming the persons they aspire to be (Eriksson, 2018). Human flourishing is, therefore, not only linked to an individual’s inner experience of the world but also to meaningful relationships with others, along with opportunities for learning and development (McCormack, 2024). Our findings underscore the importance of fostering a community that enhances staff members’ sense of belonging, where individuals are willing to cooperate and share knowledge and values as a team. On one hand, Arakelian and Rudolfsson (2023) and Dunning et al. (2021), supported this finding and highlighted that sharing the same reality among colleagues increases their sense of belonging and the meaning of their work, fostering a warm environment, a place people consider homelike which, in turn, enhances their experience of health and well-being. On the other hand, previous studies show that feelings of not belonging can lead to alienation, potentially leading to a sense of homelessness, which can threaten a person’s sense of self and contribute to suffering (Gabrielsen et al., 2014; López-Deflory et al., 2023; Ohlén et al., 2014; Salehian et al., 2023).

Like the descriptions provided by WHO (2021) and ENWHP (1997) regarding HPWE, our findings highlight the importance of engagement in developing an HPWE by fostering learning, promoting active participation, and encouraging personal development. Eriksson (2018) emphasizes that learning is fundamental to life and to individuals; it signifies development, personal growth, and engaging in a continuous process between passivity and activity in an endeavour to realize oneself and become a whole person. This finding, in line with previous studies, further emphasizes the importance of nurses’ empowerment, which is closely linked to learning and autonomy as essential factors that enhance their job satisfaction and motivation (Gu et al., 2022; Hjazeen et al., 2024; Saleh et al., 2022). Teixeira et al. (2023), in their study, found that empowerment positively impacts nurses’ satisfaction, engagement, physical and psychological health, and well-being, ultimately contributing to the quality of patient care.

The findings further highlighted that value-conscious leadership acts metaphorically as the trunk of the environmental tree, connecting the roots to the branches. It is a leadership approach and practice rooted in the core values, which serve as motivators and tone-setters, influencing the atmosphere in the environment. This finding goes beyond traditional discussions of leadership styles by emphasizing the importance of raising leaders’ consciousness of their own values and motives. This aspect is often overlooked in leadership research, yet it plays a vital role in shaping organizational culture and fostering a HPWE (James et al., 2021; Morvati & Hilli, 2023; Orvik & Axelsson, 2012; Orvik et al., 2015; Solbakken et al., 2021; Wong & Cummings, 2009). In line with previous studies, our findings underscore that nurse leaders, due to their powerful position, have a special responsibility to act as a role model and promote core values in the workplace. This includes encouraging employees to treat each other with respect, strengthening workplace relationships, facilitating learning and development, and involving employees in the decision-making process to ensure they have influence over their work (Mabona et al., 2022; Morvati & Hilli, 2023; Morvati et al., 2024; Rosengren & Friberg, 2024; Solbakken et al., 2021). Humans’ desire to take on responsibilities is deeply connected to a consciousness of their ethos (Hilli & Eriksson, 2019). Leaders who are conscious of their ethos are intrinsically motivated to foster an environment where all employees feel metaphorically “at home”—a space where everyone is respected, recognized, valued, motivated, and included (Morvati & Hilli, 2023; Morvati et al., 2024). Responsible leadership involves ethos as a reflective attitude, where leaders are aware of their position of power and use it to serve the common good—fostering employee health and well-being within a sense of community, while alleviating others’ suffering with compassion and respect (Foss et al., 2018; Morvati et al., 2024). Furthermore, consistent with earlier studies and models (Demerouti et al., 2001; Kohnen et al., 2024; Simpson et al., 2016) our findings highlight the importance of balancing demands and resources, particularly by supporting nurse leaders, ensuring sufficient staffing, and providing the necessary equipment to develop a HPWE.

## Strengths and limitations

Meta-ethnography is, through the synthesis process, a well-suited approach to gain a deeper understanding of the phenomenon of interest (Bondas & Hall, 2007; France et al., 2019). Adhering to the eMERGe reporting guidelines (France et al., 2019) enhances the transparency and clarity of the research process and serves as a quality guideline for meta-ethnography. Another notable strength is the well-balanced research team, comprising both male and female members, with expertise in qualitative research methods spanning nursing and organizational psychology.

A potential limitation is the exclusion of studies published in languages other than English and Scandinavian, which may lead to overlooking current and relevant studies on HPWE. However, the meta-ethnographic approach does not aim to summarize all pertinent literature but seeks conceptual insight, requiring a sufficient number of studies to illuminate the phenomenon and facilitate the translation process (Campbell et al., 2011; Thorne et al., 2004). The included studies represented different contexts and countries. On the one hand, this may strengthen the transferability of the findings to various contexts, as they contribute to a rich data material. However, on the other hand, it could also be considered a limitation (France et al., 2019). According to Sandelowski and Barroso (2007), meta-synthesis can be criticized for not adequately considering the unique experiences of the original findings and for detaching them from their context. We were aware of this during the synthesis phases. However, meta-ethnography aims to develop a new overarching understanding of the phenomenon of interest. Its focus is to go beyond the aggregation of findings to integrate and interpret the results at a higher level (France et al., 2019; Noblit & Hare, 1988; Thorne et al., 2004). In the interpretation and synthesis phases, there is a risk of either not delving deep enough into the data interpretation, leading to a more descriptive summary of previous findings, or over-interpreting the data, potentially disconnecting them from the original studies (Bondas & Hall, 2007; France et al., 2019). To address this limitation, the researchers actively participated in all phases of the meta-ethnography. We held regular meetings and conducted in-depth and reflective discussions focused on refining the interpretation and synthesis processes. Through these discussions, the researchers reached a consensus on the synthesis and interpretation of the data, which in turn led to the transparency and rigour of the research process. The included studies represented nurses’ perspectives more than leaders on a HPWE, which led to an imbalance between the two perspectives and potentially influenced the findings. However, this imbalance may reflect reality, where the number of nurses is higher than that of nurse leaders.

## Conclusions

This meta-ethnography provides a deeper understanding of a health-promoting work environment that can guide the development of an environment which may enhance nurses’ health, well-being, and job satisfaction. Nurses and nurse leaders interact with each other in day-to-day work; therefore, collaboration between them is essential for fostering an environment where everyone can feel respected, recognized, valued, and involved. However, leaders, due to their power positions, have a special responsibility to facilitate a HPWE by implementing, among others, training and learning programmes, encouraging reflection on shared values in the workplace, involving nurses in decision-making processes, and ensuring a balance between workload and staffing. Consequently, leaders need to receive adequate resources and support from their superiors to foster an environment that enhances nurses’ health and job satisfaction. Given the importance of nurse leaders’ role and consciousness of their values and their impact on the work environment, future research should focus on understanding the values that motivate leaders and the action strategies they employ in developing a HPWE.

## Supplementary Material

Supplemental Material

## Data Availability

The data that support the findings of this study are available from the corresponding author upon reasonable request.
