# Supplementary material for: Nurses’ and nurse leaders’ perspectives on a health-promoting work environment: a meta-ethnographic study
Source: Int J Qual Stud Health Well-being. 2025 Jan 31;20(1):2460255. doi: 10.1080/17482631.2025.2460255 (PMC11789224; doi:10.1080/17482631.2025.2460255)
Supplement: Supplemental Material [file ZQHW_A_2460255_SM7023.docx]

**Appendix**

**Figure S1 A)** ROC curve for MRC detecting reduced LVEF**. B)** ROC curve for MFAP4 detecting reduced LVEF**.**


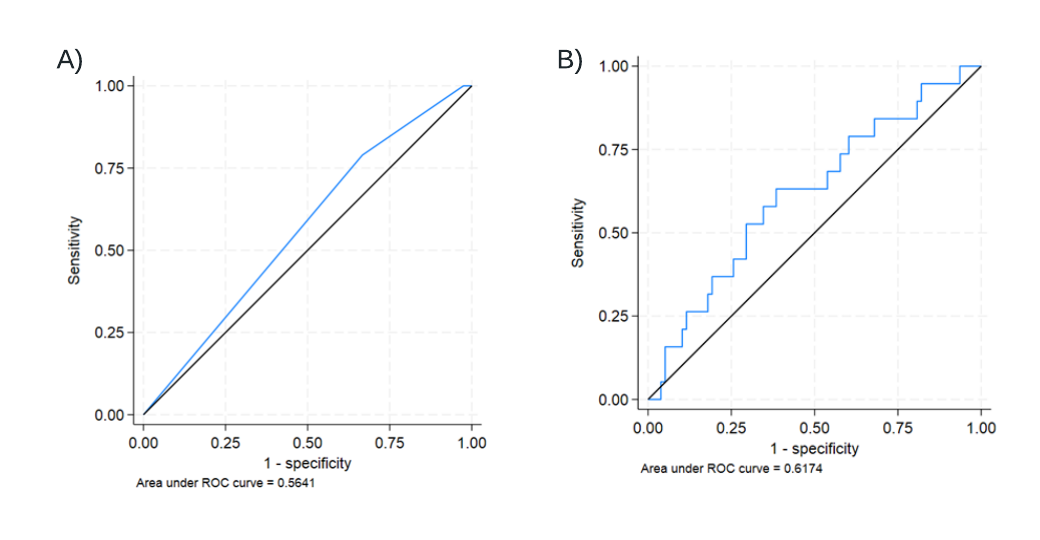


Figure S1 legend: ROC= Receiver operator curve. MRC= The Medical Research Council dyspnoea scale (range 1-5), MFAP4=Microfibrillar-associated protein 4.

| Table S1: Diagnostic outcome measures for detecting LVEF <50% | | |
| --- | --- | --- |
|  | MRC ≤0  n=≤ (0%) | MFAP4 ≥ 15.9  n=94 (97%) |
| LVEF <50%  n= 19 | N/A | 19 (100%) |
| LVEF ≥50%  n= 78 | N/A | 75 (96%) |
| Diagnostic OR (95% CI) | N/A | 2.22 (1.10; 4.50) |
| Sensitivity, % (95% CI) | N/A | 62.7 (49.1; 75.0) |
| Specificity, % (95% CI) | N/A | 56.9 (44.7; 68.6) |
| PPV, % (95% CI) | N/A | 54.4 (41.9; 66.5) |
| NPV, % (95% CI) | N/A | 65.1 (52.0; 76.7) |

Table S1: MRC= The Medical Research Council dyspnoea scale, MFAP4=Microfibrillar-associated protein 4, n=number, N/A=Not applicable, CI=confidence interval, PPV=Positive predictive value, NPV=Negative predictive value.
